# Nanoscale quantification of the biophysical characterization of combretastatin A-4-treated tumor cells using atomic force microscopy

**DOI:** 10.1371/journal.pone.0179115

**Published:** 2017-06-19

**Authors:** Yanchun Li, Jv Chen, Yutong Liu, Weige Zhang, Wenhui He, Hanying Xu, Lianqing Liu, Enlong Ma

**Affiliations:** 1 Department of Pharmacology, Shenyang Pharmaceutical University, Shenyang, China; 2 State Key Laboratory of Robotics, Shenyang Institute of Automation, China Academy of Sciences, Shenyang, China; 3 Department of Medicinal Chemistry, Shenyang Pharmaceutical University, Shenyang, China; LAAS-CNRS, FRANCE

## Abstract

As an inhibitor of microtubule assembly, combretastatin A-4 (CA-4)-induced biological responses in tumor cells have been well known, but the corresponding changes in nano-biophysical properties were not investigated given the lack of an ideal tool. Using AFM technique, we investigated the alteration of nano-biophysical properties when CA-4-treated tumor cells underwent the different biological processes, including cell cycle arrest, apoptosis and autophagy. We found that CA-4-resistant cells were rougher with the presence of characteristic “ridges”, indicating that the development of “ridge” structure may be a determinant of the sensitivity of cells to CA-4 compounds. CA-4 induced G2/M arrest and apoptosis in sensitive cells but triggered anti-apoptotic autophagy in resistant cells. CA-4 treatment caused an increase in stiffness in both sensitive and resistant cells. However, these cells exhibited different changes in cell surface roughness. CA-4 decreased Ra and Rq values in sensitive cells but increased these values in resistant cells. The reorganization of F-actin might contribute to the different changes of nano-biophysical properties in CA-4-sensitive and–resistant cells. Our results suggest that cellular nano-biophysical properties, such as “ridges”, roughness and stiffness, could be applied as potential biomarkers for evaluating CA-4 compounds, and knowledge regarding how biological alterations cause changes in cellular nano-biophysical properties is helpful to develop a new high-resolution screening tool for anti-tumor agents.

## Introduction

Combretastatins are a class of anti-mitotic agents isolated from the bark of the South African tree *Combretum caffrum* [1]. Combretastatin A-4 (CA-4) is most active among them and exhibits potent anti-proliferative activity against a wide spectrum of tumor cells by inhibition of tubulin polymerization. CA-4 together with its water-soluble prodrug combretastatin A-4 phosphate (CA-4P) are currently undergoing clinical trials for the treatment of various solid tumors. What makes this class of compounds more interesting than other anti-mitotic agents is that they also exhibit anti-angiogenic effects. These compounds bind to the colchicine binding site of β-tubulin and lead to depolymerization of microtubules. As a vascular-disrupting agent (VDA), CA-4 selectively blocks or destroys the pre-existing blood vessels in tumor tissue, leading to rapid shutdown of the blood supply in tumor tissue and subsequent killing of tumor cells via oxygen and nutrient deprivation [2, 3].

The cytoskeleton is a complex polymeric network, and its dynamic characteristics determine the variety of cell shape and mechanical properties. Alterations of the cytoskeleton structure are often induced by different biological responses [4]. Given that tumor progression is characterized by disruption and/or reorganization of the cytoskeleton, further leading to alterations of the cytoarchitecture and biomechanical properties, cyto-biophysical properties may serve as biomarkers for evaluating the efficacy of anti-tumor agents [5], especially those that function by affecting the assembly of tubulin and the cytoskeleton. CA-4 compounds exhibit powerful anti-tumor activity by influencing cell microtubules and changing the cytoskeleton structure; however, how these changes affect the nanostructure and nanomechanics of tumor cells are unknown. At present, the approaches for assessing CA-4 compounds mainly rely on classical biological assays, but these methods can not directly reflect (visualize) the changes in the cytoskeletal structure and cyto-biomechanical properties. The presence of atomic force microscopy (AFM) meets the demand for visualizing the cyto-biophysical properties.

In 1986, Binnig et al developed the atomic force microscope, a technique that allowed the visualization of the cell surface on an atomic scale. AFM is a powerful, versatile and easy-to-control nanometric imaging technique for investigating the cyto-biophysical properties at a single molecular level [6, 7]. This technology provides surface morphology, structure and biomechanics of cells at nanoscale resolution under near-physiological conditions, allowing researchers to detect cellular nano-biophysical properties and better understand the relation between cell biology and cyto-biophysical characteristics [8–10]. Using a visualized AFM technique, the goal of the present study is to investigate the alteration of nano-biophysical properties when CA-4-treated tumor cells undergo different biological processes, including microtubule depolymerization, cell cycle arrest, cell apoptosis and autophagy. Knowledge regarding how biological alterations cause changes in nano-biophysical properties is helpful for developing a new high-resolution screening tool for anti-tumor agents.

## Materials and methods

### Reagents

Combretastatin A-4 was provided by Prof. Weige Zhang (Department of Medicinal Chemistry, Shenyang Pharmaceutical University, China). RPMI 1640 medium with L-glutamine, Hanks Balanced Salt Solutions (HBSS), polylysine, fetal bovine serum, penicillin G and streptomycin was obtained from GIBCO BRL (Gaithersburg, MD). Actin Tracker Green and Tubulin Tracker Red were obtained from Beyotime Institute of Biotechnology (Naijing, China). Dimethyl sulfoxide (DMSO), ribonuclease (RNase), propidium iodide (PI), MTT (3-(4,5-dimethylthiazol-2-yl)-2,5-diphenyltetrazoliumbromide), monodansylcadaverine (MDC), and acridine orange (AO) were purchased from Sigma Chemical (St. Louis, MO).

### Cell culture

HepG2 human hepatic tumor cells, HeLa human cervical tumor cells, and MCF-7 human breast tumor cells were cultured in RPMI 1640 medium supplemented with 10% (v/v) heat-inactivated fetal bovine serum (FBS), 1 mM-glutamine, 100 U/ml penicillin and 100 μg/ml streptomycin at 37°C in 100% humidity and 5% CO_2_.

### MTT assay

The growth inhibitory effect of CA-4 *in vitro* was measured by the MTT assay. Cells seeded in 96-well plates were treated with different concentrations of CA-4. DMSO (end-concentration of 0.1%) was used as a control group. After 24, 48 and 72 h of incubation, 5.0 mg/ml MTT solution was added, and the plates were incubated for an additional 4 h at 37°C. The purple formazan crystals were dissolved in 100 μl DMSO, and the plates were read on an ELISA reader at 570 nm. The cell viability was calculated as the ratio of the absorbance of treated cells to the absorbance of the control groups. IC_50_ values were calculated using the Statistical Product and Service Solutions software. All experiments were performed in triplicate in three independent experiments.

### Flow cytometric analysis for cell cycle distribution

Cell cycle distribution was detected using flow cytometry. HepG2, HeLa and MCF-7 cells (1×10^6^ cells) were incubated with various concentrations of CA-4 or 0.1% DMSO for the indicated times. After harvesting by trypsinization, the cells were washed with PBS and fixed in ice-cold 70% (v/v) ethanol. The fixed cells were harvested by centrifugation and resuspended in 500 μl of PBS containing 100 μg/ml RNase. After a 30-min incubation at 37°C, the cells were stained with 50 μg/ml PI at 4°C in the dark for 30 min. Flow cytometry was then performed on a FACScan, with collection and analysis of data using Cell Quest software.

### AO and MDC staining for autophagy detection

The presence of the cellular acidic compartment served as a marker to determine autophagy after AO staining. MCF-7 cells were seeded in 24-well and exposed to various concentrations of CA-4 for the indicated times. After incubation with medium containing 1 mg/ml AO for 15 min, the fluorescent micrographs were collected using an inverted fluorescent microscope. MDC staining was used to confirm the abundance of autophagic vacuoles in cells. CA-4-treated MCF-7 cells were stained with 50 μM MDC for 1 h at 37°C, and then examined by fluorescence microscopy or analyzed on a flow cytometer using Cell Quest software [11, 12].

### AFM imaging

Morphological and ultrastructural assays were performed using a Nanoscope VI Dimension 3100 AFM (Veeco Company, Santa Barbara, CA) and oxide-sharpened Si3N4 tips (MLCT, radius 10–40 nm; Bruker Company, Santa Barbara, CA). Cells were seeded in 70-mm culture disks. For live cell imaging, the triangular cantilever with a normal spring constant of 0.01 N/m was employed. The spring constant was calibrated with a Thermal Tune Adapter (Veeco Company). The probe was localized on the cell surface with the assistance of a CCD camera. The experiments were performed at contact mode. The scan force was 50 pN, and the scan rate was 0.3 Hz. The Nanoscope Analysis software 1.50sr3 (Bruker) was used to process AFM images and obtain the Mean Roughness (Ra) and the Root Mean Square roughness (Rq). Ra and Rq were calculated and averaged from four identical scan areas (3 μm × 3 μm) [13].

### Measurement of mechanical properties by AFM

The elasticity of cells was also detected at contact mode. Measurements were conducted above the nuclear region of the cell to avoid the influence of the underlying substrate. Then, 100 Force-distance curves were obtained from 10 different cells in each group at the ramp rate of 0.5 Hz. Force curves were obtained at the same loading rate and were analyzed by Matlab 7.6.0. The Hertz model was used to compute the Young’s modulus according to the following formulas (F: loading force, v: Poisson ratio, δ: indentation, E: Young’s modulus, R: radius of the curvature of the AFM tip) [13].

Fsphere=4ER12S323(1−υ2) (1)

$$F_{cone}=\frac{2E\delta^{2}\text{tan}\theta }{\pi \left(1-\upsilon^{2}\right)}$$ (2)

### Fluorescence imaging of cytoskeletal proteins

The cells (1 × 10^4^) were plated with a glass cover slips in 24-well plate and incubated with CA-4 for the indicated times. After treatment, the cells were fixed with 3.7% formaldehyde in PBS for 10 min, washed twice with PBS and permeabilized in 0.1% Triton X-100 in PBS for 10 min at room temperature. The cells were then stained with 1:200 Actin-Tracker Green (phalloidin-FITC) and Tubulin-Tracker Red (a-Tubulin-Alexa Fluor 555), separately. Finally, the cytoskeletal proteins were imaged using Olympus FluoView^™^ FV1000 Confocal Microscope.

### Statistical analysis

The results are expressed as the mean ± SD. The difference between multiple groups was assessed by one-way analysis of variance (ANOVA) followed by Scheffé’s multiple range tests. *P<0.05, **P<0.01, compared with the control group.

## Results

### Biological responses induced by CA-4 in various tumor cells

In the present study, we chose three representative tumor cells (HepG2, HeLa, and MCF-7 cells) for all tests that exhibited high, moderate and low sensitivity to CA-4, respectively, according to preliminary findings (S1 Fig). The anti-growth effects of CA-4 on the three types of cells were assessed by MTT assays. After 72 h of CA-4 treatment, the IC_50_ values for HepG2, HeLa and MCF-7 cells were 6.5, 17.3 and 188.2 nM, respectively.

**Fig 1:**
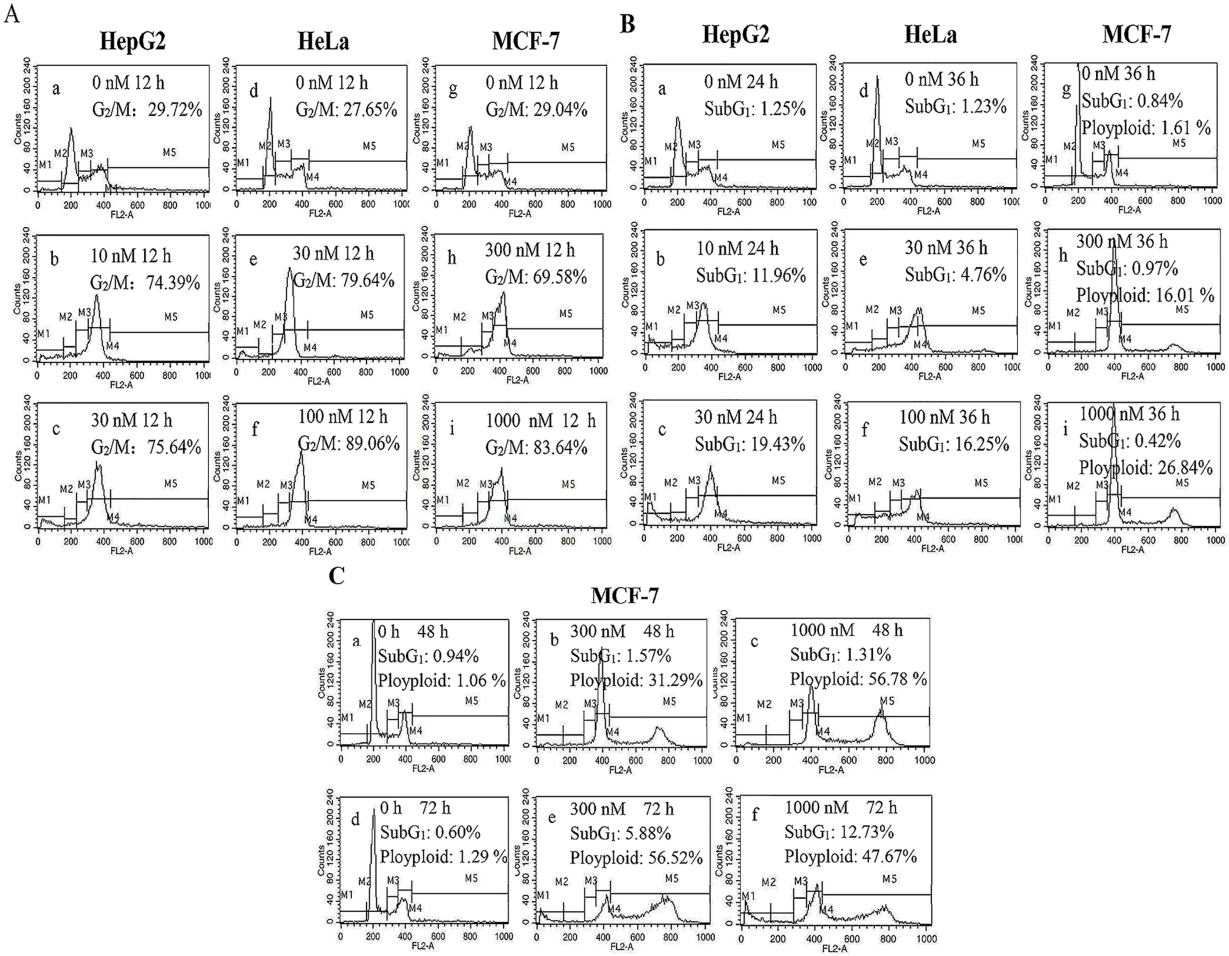
Biological responses induced by CA-4 in HepG2, HeLa and MCF-7 cells. (A) CA-4 induced G2/M phase arrest. HepG2 (a-c), HeLa (d-f) and MCF-7 (g-i) cells were treated with different concentrations of CA-4 for 12 h, and the cells were then stained with PI and subjected to flow cytometric analysis. (B) Prolonged treatment of CA-4 induced apoptosis in HepG2 and HeLa cells but not in MCF-7 cells. HepG2 (a-c), HeLa (d-f) and MCF-7 (g-i) cells were treated with different concentrations of CA-4 for 24 to 36 h, and the cells were then stained with PI and subjected to flow cytometric analysis. (C) CA-4 induced the presence of DNA polyploidy in MCF-7 cells before inducing apoptosis. MCF-7 cells were treated with CA-4 (300 and 1000 nM) for 48 (a-c) or 72 (d-f) h, and the cells were then stained with PI and subjected to flow cytometric analysis.

To investigate the mechanisms of action of CA-4 in tested cells, we studied the influence of CA-4 in cell cycle distribution by flow cytometry. HepG2, HeLa and MCF-7 cells were treated with various concentrations of CA-4 according to their sensitivity to CA-4. After 12 h of treatment, CA-4 induced G2/M phase arrest in all tested cells, but a higher concentration of CA-4 (300 nM) was required to produce obvious G2/M arrest in MCF-7 cells compared with HepG2 and HeLa cells (Fig 1A).

Previous studies have demonstrated that microtubule-inhibiting agents caused G2/M phase arrest and then triggered apoptosis [14, 15]. We therefore prolonged the treatment time of CA-4 to assess whether apoptosis occurred. Here, 30 nM of CA-4 induced apoptosis in HepG2 cells after 24 h of treatment as demonstrated by an increase in the percentage of sub-G1 cells from 1.25% to 17.96% (Fig 1B). A higher concentration of CA-4 (100 nM) and longer treatment time (36 h) are needed to produce apoptosis-inducing effects in HeLa cells (Fig 1B). However, 1000 nM of CA-4 did not induce the presence of the sub-G1 peak in MCF-7 cells, even when treatment time was prolonged to 48 h (Fig 1B). Interestingly, we observed the presence of DNA polyploidy (≥4N DNA content) in CA-4-treated MCF-7 cells. With the prolonged treatment of CA-4, the percentage of the polyploidy peak gradually increased, and 36 and 48 h of CA-4 treatment (300 nM) resulted in an increase in the percentage of the polyploidy peak from 1.51% to 16.01% and 31.29%, respectively (Fig 1C). In addition, the percentage of MCF-7 cells in the sub-G1 phase increased until 72 h of CA-4 treatment (Fig 1C).

### Nano-morphological and ultrastructural changes in tumor cells undergoing cell cycle arrest and apoptosis

**Fig 2:**
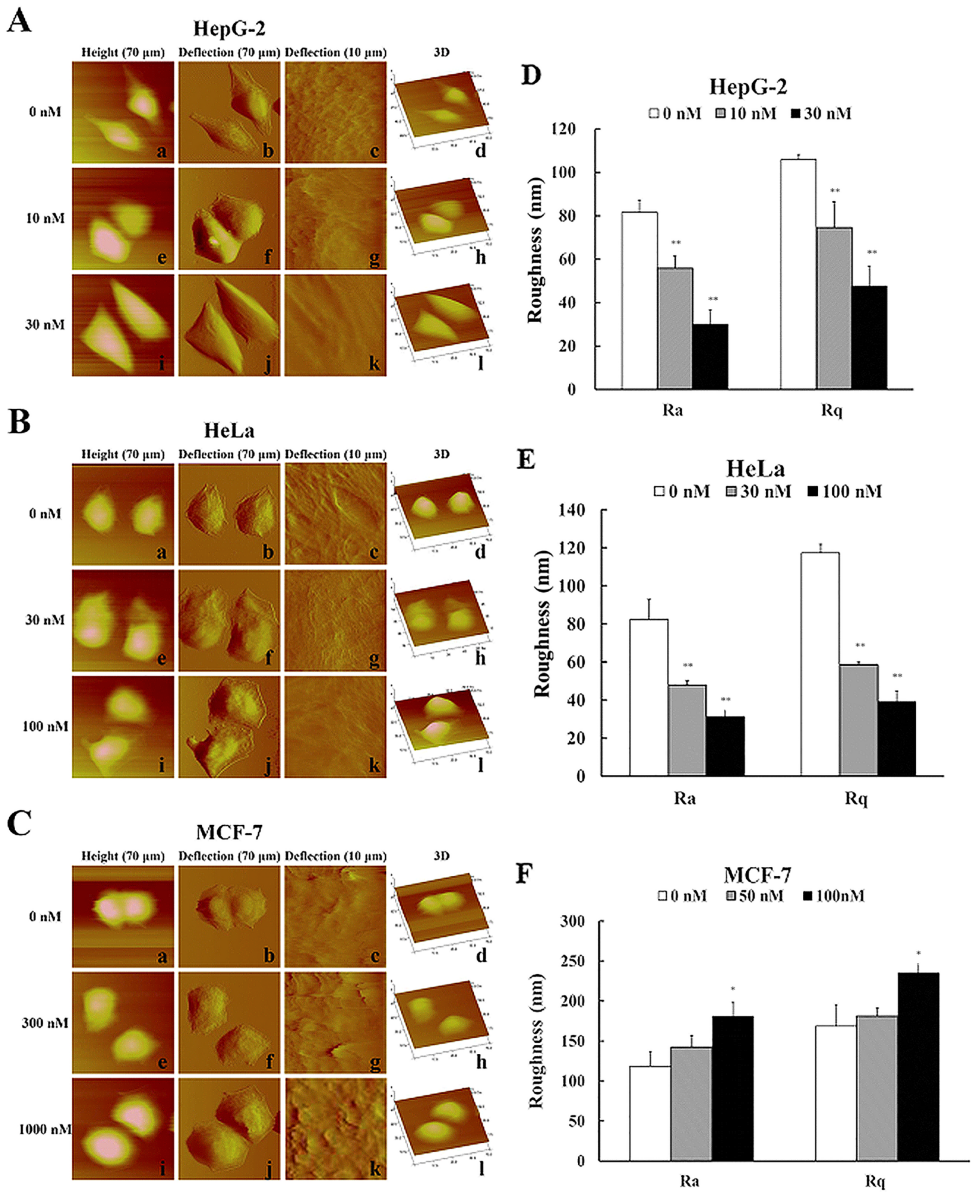
Nanoscale morphological and ultrastructural changes in tumor cells undergoing cell cycle arrest. HepG2 (A, D), HeLa (B, E) and MCF-7 (C, F) cells were treated with different concentrations of CA-4 for 12 h, and the cells were then imaged by AFM. (a), (e) and (i) in each picture show height images of cells; (b), (f), (j) and (c), (g), (k) show deflection images of 70 × 70 μm and 10 × 10 μm; (d), (h) and (i) are 3D images of cells. (D), (E) and (F) are quantified results of Ra and Rq values in the tested cells, n = 6. The scale bars “-” and “⋯” are 10 μm and 1 μm, respectively.

The living cells in Hanks solution were imaged to evaluate the nano-morphological and ultrastructural changes induced by CA-4. As demonstrated in Fig 2A–2C, all untreated cells displayed typical elliptical shape and clear boundaries. Compared with the cytoskeleton of HepG2 and HeLa cells, the MCF-7 cell surface was rougher and decorated with numerous dorsal “ruffles” or “ridges”. Furthermore, local cell imaging by AFM revealed disruption of the cellular cytoskeleton network and alterations in the Ra and Rq values (markers of cell surface roughness) of the cell surface after 12 h of CA-4 treatment (G2/M arrest). Interestingly, each type of cell produced different changes in the Ra and Rq values. CA-4 decreased Ra and Rq values in HepG2 and HeLa cells (Fig 2D–2F), suggesting that these cells tended to have a smooth cell surface after CA-4 treatment. Unlike HepG2 and HeLa cells, CA-4 increased Ra and Rq values in MCF-7 cells, indicating that the surface of MCF-7 cells became rougher. The 3D AFM imaging figures were consistent with the above results.

**Fig 3:**
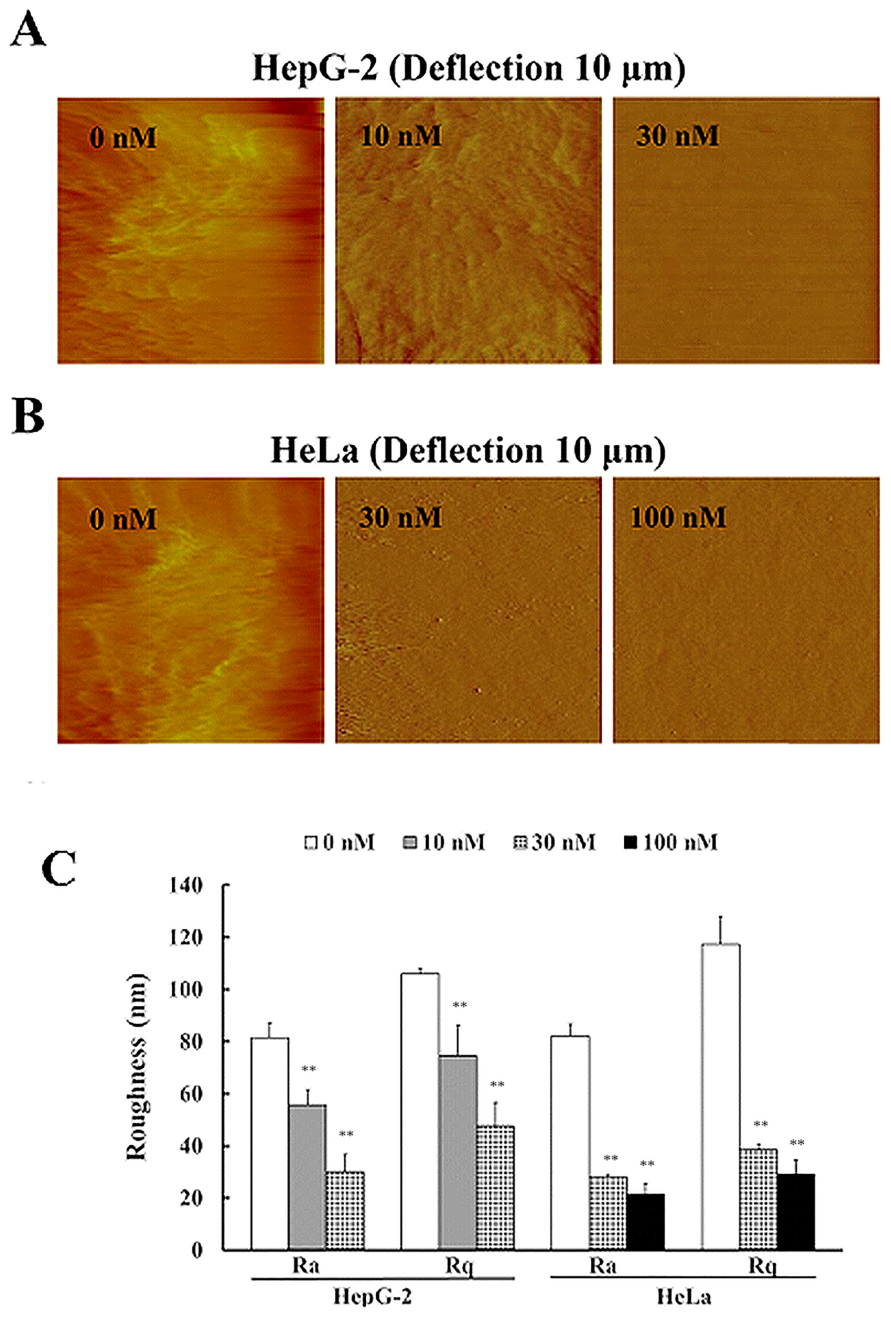
Nanoscale morphological and ultrastructural changes in tumor cells undergoing apoptosis. The cells were treated with different concentrations of CA-4 for 24 or 36 h and then imaged by AFM. (A) and (B) are deflection images of 10×10 μm for HepG2 and HeLa cells, respectively. (C) is the quantified results of Ra and Rq values in the tested cells, n = 6. The scale bars are 1 μm.

We continued to investigate the nano-morphological and ultrastructural changes in tumor cells undergoing apoptosis. Local cell AFM imaging revealed that cytoskeletal structure was almost completely destroyed and that Ra and Rq values were further reduced when HepG2 and HeLa cells underwent apoptosis after 24 or 36 h of CA-4 treatment (Fig 3).

### Biomechanical changes in tumor cells undergoing cell cycle arrest and apoptosis

**Fig 4:**
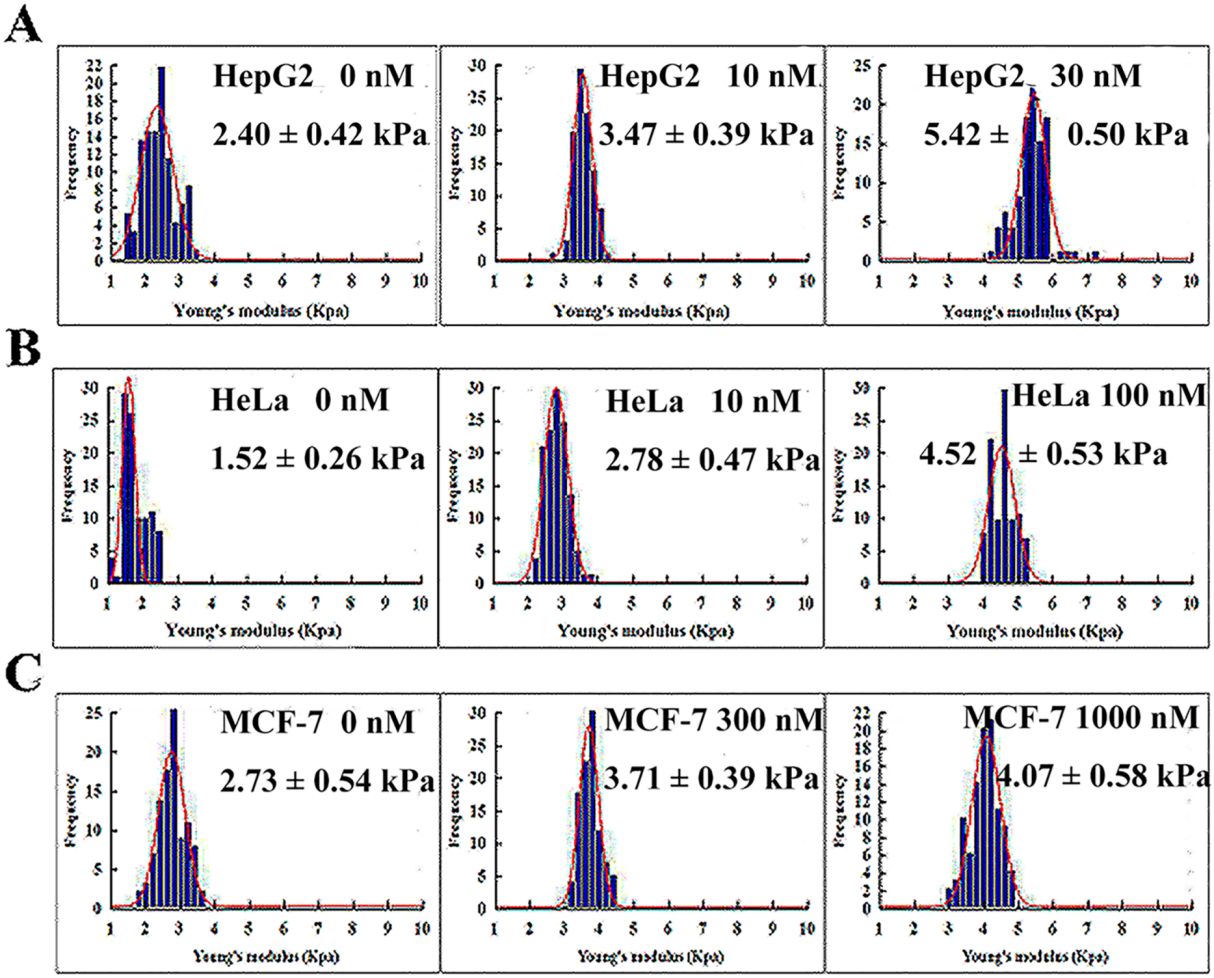
Nanoscale biomechanical changes in tumor cells undergoing G2/M arrest. HepG2, HeLa and MCF-7 cells were treated with CA-4 at the indicated concentrations for 12 h, and Young's modulus was then detected by AFM. (A), (B) and (C) are representative experiments for HepG2, HeLa and MCF-7 cells, respectively, n = 10.

**Fig 5:**
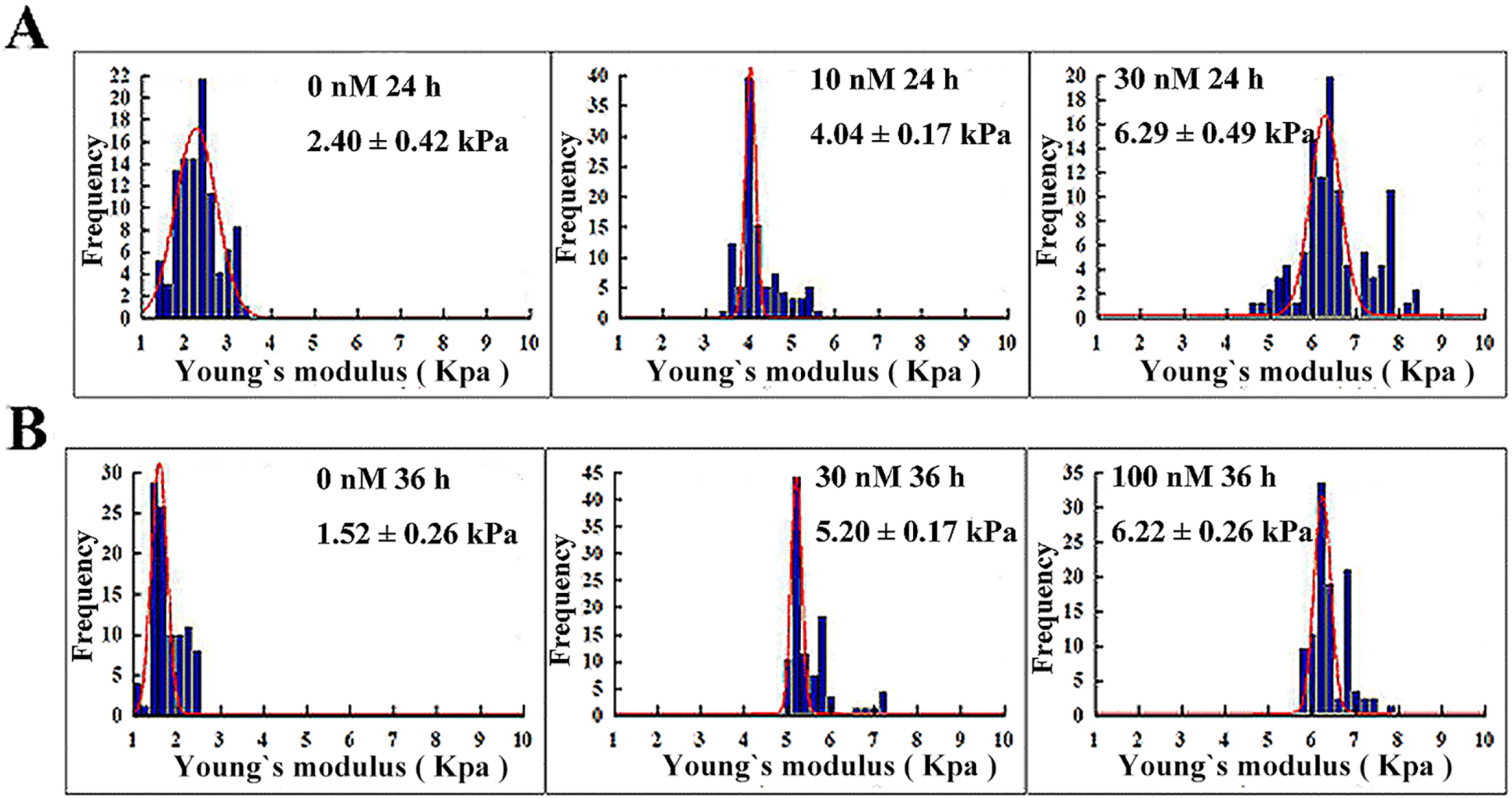
Nanoscale biomechanical changes in tumor cells undergoing apoptosis. HepG2 and HeLa cells were treated with CA-4 at the indicated concentrations for 12 or 36 h, and Young's modulus was then detected by AFM. (A) and (B) are representative experiments for HepG2 and HeLa cells, n = 10.

We also used AFM to investigate the influence of CA-4 on cyto-biomechanical properties. When cells were stimulated with different concentrations of CA-4 for 12 h to induce G2/M arrest, an increased Young’s modulus was observed in all tested cells (Fig 4), indicating that the biological response of G2/M phase arrest was accompanied by an increase in cell stiffness. Among the three tumor cells, CA-4-sensitive HepG2 cells produced the most significant changes in Young’s modulus, but CA-4-resistant MCF-7 cells exhibited less change in Young’s modulus. Furthermore, apoptotic HepG2 and HeLa cells were significantly stiffer when the exposure time of cells to CA-4 was extended to 24 or 36 h (Fig 5).

### Autophagy and corresponding ultrastructural and biomechanical changes in CA-4-treated MCF-7 cells

**Fig 6:**
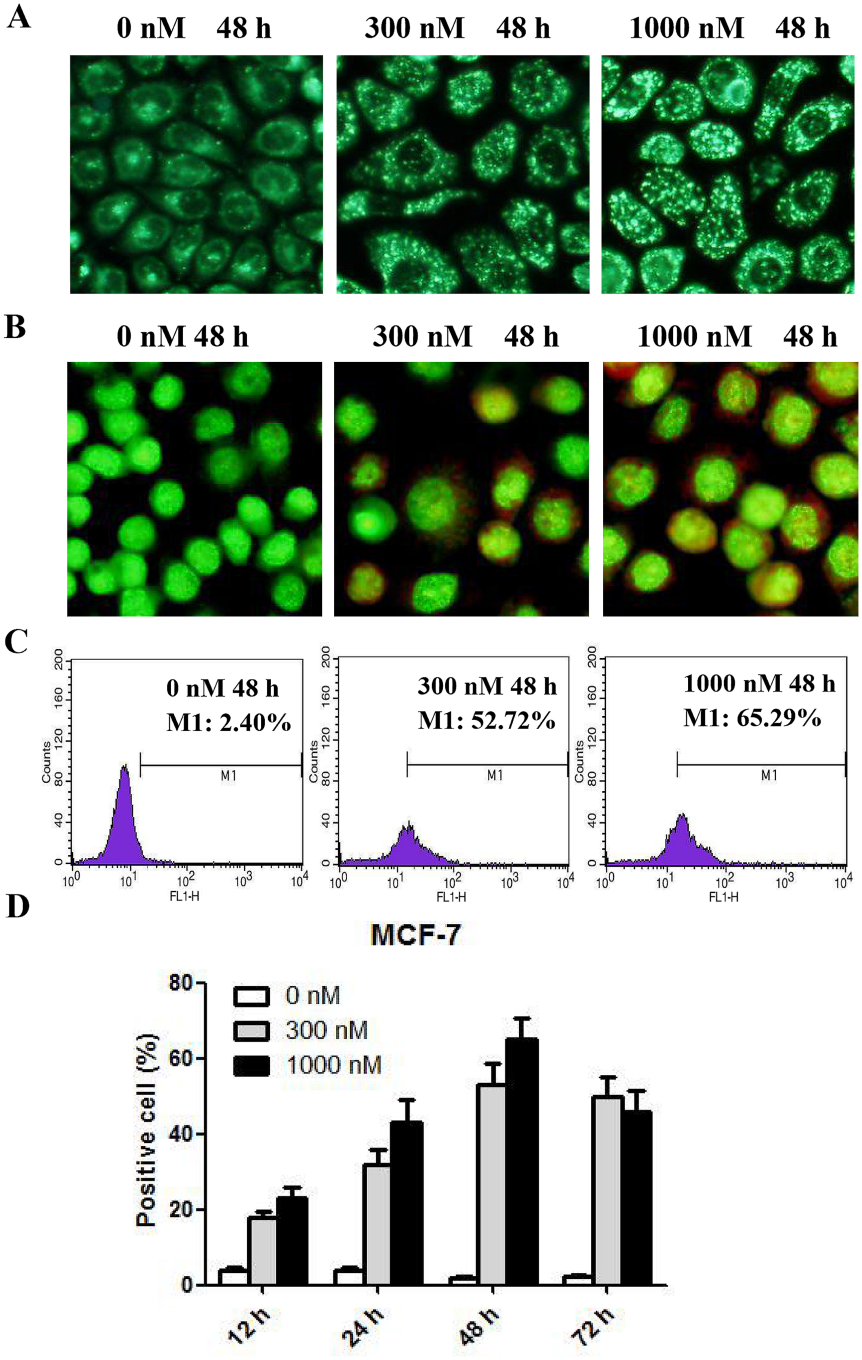
CA-4 induced autophagy in MCF-7 cells. Autophagosome observation: MCF-7 cells were treated with CA-4 at the indicated concentrations for 48 h. After treatment, the cells were stained with MDC (A) or AO (B) and then observed by a fluorescence microscope. Autophagic cell detection: MCF-7 cells were treated with CA-4 at the indicated concentrations for 12, 24, 48 or 72 h. After treatment, the cells were stained with MDC, and then concentration- and time-dependent autophagy-inducing effects (C and D) were detected by flow cytometry, n = 3.

Given that CA-4 did not trigger obvious apoptosis in MCF-7 cells, we tested whether CA-4 could induce anti-apoptotic autophagy. The development of acidic vesicular organelles (AVOs) in cytoplasm is a characteristic of autophagy [12, 16]. To assess the development of AVOs, the cells were treated with MDC and AO staining of autophagic vacuoles after CA-4 treatment. As shown in Fig 6A (MDC staining) and Fig 6B (AO staining), AVOs were observed in MCF-7 cells after exposure to CA-4 for 48 h. To quantify AVOs, we used flow cytometry to detect the MDC fluorescence intensity. As demonstrated in Fig 6C and 6D, when MCF-7 cells were treated with 300 or 1000 nM CA-4 for 12, 24, 48 and 72 h, the percentage of autophagic cells increased in a time- and concentration-dependent manner, indicating the autophagy-inducing effect of CA-4 in MCF-7 cells.

**Fig 7:**
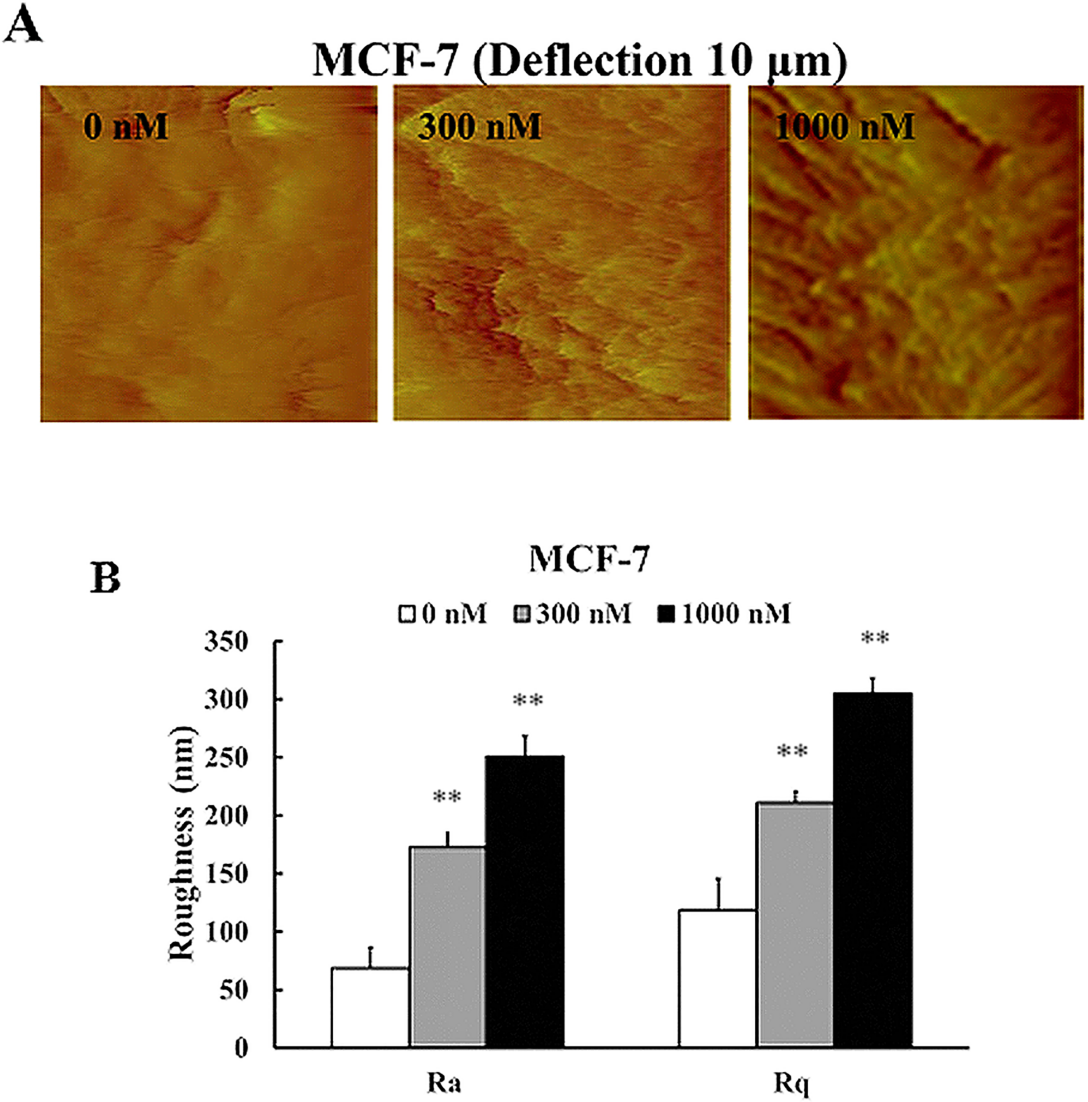
Nanoscale morphological and ultrastructural changes in autophagic MCF-7 cells. MCF-7 cells were treated with CA-4 at the indicated concentrations for 48 h, and the cells were then imaged by AFM. (A) Deflection images of 10 × 10 μm. (B) Quantified results of Ra and Rq values, n = 6. The scale bars are 1 μm.

**Fig 8:**
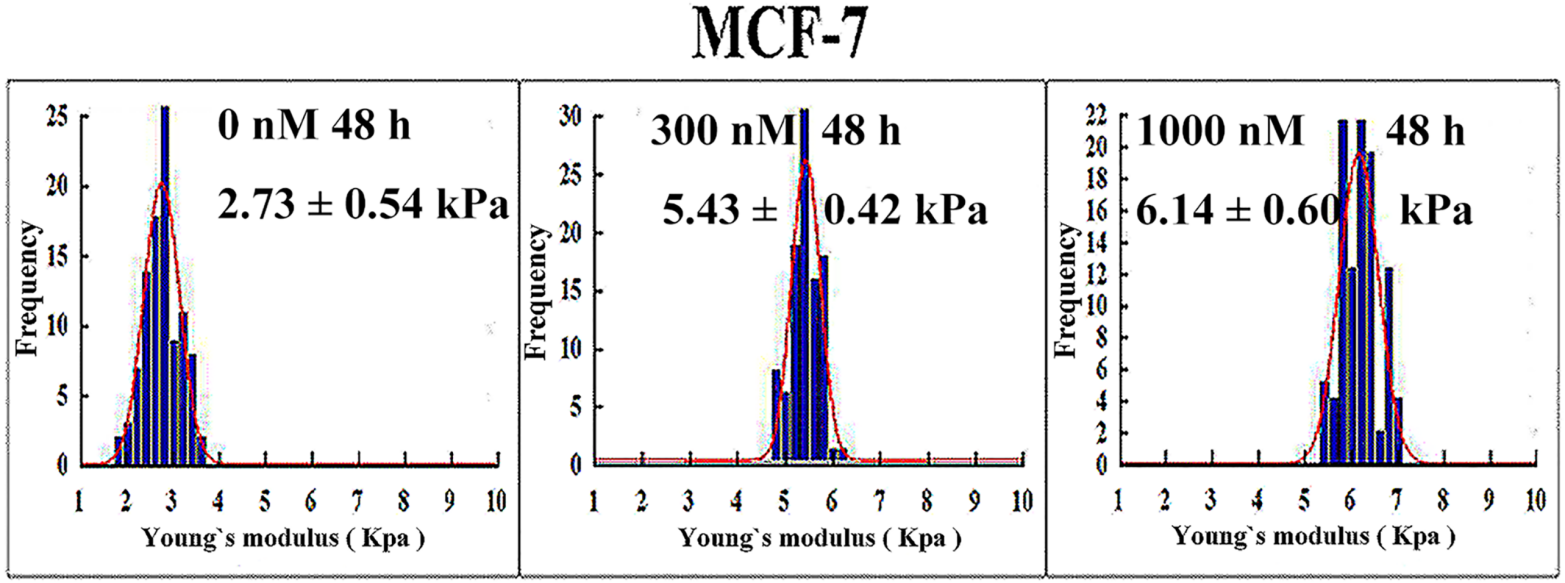
Nanoscale biomechanical changes in autophagic MCF-7 cells. MCF-7 cells were treated with CA-4 at the indicated concentrations for 48 h, and Young's modulus was then detected by AFM, n = 10.

AFM was then used to detect the nano-biophysical changes in autophagic MCF-7 cells. As shown in Fig 7, after treatment with CA-4 for 48 h, autophagic MCF-7 cells had obvious “ridges” and higher Ra and Rq values (roughness) on their membrane surface. The biomechanical detection showed that 300 and 1000 nM CA-4 induced an increase in the Young’s modulus in MCF-7 cells from 2.73 to 5.43 and 6.14 kPa, respectively (Fig 8), suggesting that the autophagic MCF-7 cells also became stiffer. However, the changes in the cytoskeleton network and Young’s modulus in the autophagic MCF-7 cells were less than that in apoptotic HepG2 and HeLa cells.

### The reorganization of cytoskeleton in CA-4-treated tumor cells

**Fig 9:**
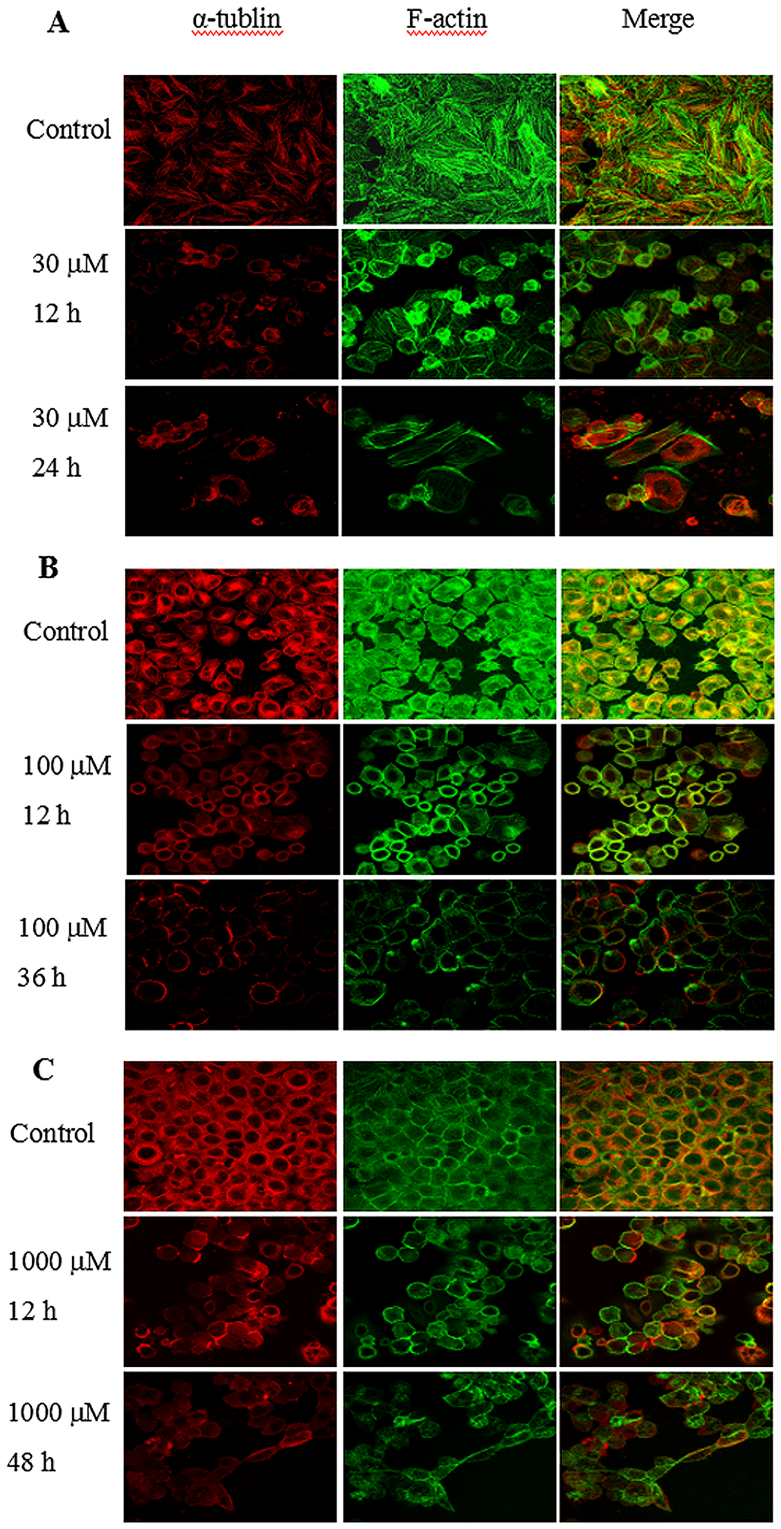
Fluorescence imaging of cytoskeletal proteins α-tublin and F-actin in CA-4-treated tumor cells. HepG2 (A), HeLa (B) and MCF-7 (C) cells were treated with CA-4 for indicated times, and then stained with Actin-Tracker Green and Tubulin-Tracker Red, separately. The cytoskeletal proteins were imaged using Olympus FluoView^™^ FV1000 Confocal Microscope. The scale bar is 20 μm.

The effect of CA-4 on the cytoskeleton was investigated by fluorescence imaging of cytoskeletal proteins α-tublin and filamentous actin (F-actin). As shown in Fig 9, α-tublin (red fluorescence) of all untreated cells was equally distributed into cytoplasma. After 12 h of CA-4 stimulation (inducing G2/M arrest), the decreased fluorescence intensity in cytoplasma was observed in all tested cells. With prolongation of treating times (inducing apoptosis or autophygy), the fluorescence intensity in cytoplasma further decreased. We consider that this reorganization of α-Tublin induced by CA-4 should be due to depolymerization of microtubules. As for F-actin regulating morphology and biomechanics of cells, the green fluorescence can be detected throughout the whole untreated cells. The distribution of F-actin in CA-4-sensitive HepG-2 and HeLa cells was almost equal, but denser fluorescence intensity was observed in cytoplasmic membrane of MCF-7 cells. 12 h of CA-4 treatment induced the reorganization and polymerization of F-actin into cytoplasma in both CA-4-sensitive and -resistant tumor cells. The prolonged treatment of CA-4 resulted in further polymerization of F-actin in CA-4-sensitive HepG-2 and HeLa cells. In contrast, F-actin in MCF-7 cells fragmented and unequally distributed into the whole cells. According to the results above, F-actin was reorganized in different ways, when CA-4-stimulated tumor cells underwent G2/M arrest, apoptosis or autophygy. Therefore, the reorganization of F-actin might contribute to the different changes of nano-biophysical properties in CA-4-sensitive and—resistant cells.

## Discussion

The microtubule network plays an important role in supporting the cytoskeleton structure and biomechanical properties of living cells. As a microtubule assembly inhibitor, CA-4 can cause cellular microtubule depolymerization and induce G2/M phase arrest [17]. Although the biological responses induced by CA-4 are well known, the corresponding ultra-microstructural and biomechanical changes were not investigated given the lack of a visualization tool. Given that separation of cytotoxic activity from the ability to inhibit the assembly of tubulin is considered a realistic goal in the synthesis of CA-4 analogues [18], it is more interesting to understand the influence of CA-4 in cellular nano-biophysical properties, including nano-morphology, biomechanics and cytoskeleton structure, at the single cell level.

Using AFM nanometric imaging techniques, we found that the cytoskeleton of CA-4-sensitive HepG2 and HeLa cells was more organized, whereas the surface of resistant MCF-7 cells was very rough and presented with numerous dorsal “ruffles” or “ridges”. An obvious “ridge” structure and a rougher surface were also noted in U251 and L929 cells with lower sensitivity to CA-4 (S2 Fig). Given that the ridges (or ruffles) were constantly detected only on CA-4-resistant tumor cells, we assume that the presence of “ridges” might be related to the sensitivity of cells to CA-4.

Biological assays showed that CA-4 exhibited more potent anti-proliferative activity against HepG2 and HeLa cells by inducing G2/M arrest and apoptosis. We also investigated the corresponding alteration of nano-biophysical properties in these cells. Twelve hours of CA-4 treatment arrested the cell cycle at G2/M phase, and lower surface roughness and higher cell stiffness were observed in CA-4-treated HepG2 and HeLa cells. The prolonged treatment of CA-4 from 12 to 36 h resulted in an accumulation of HepG2 and HeLa cells in the sub-G1 phase, suggesting that CA-4-stimulated cells underwent apoptosis following G2/M phase arrest. When cells were in an apoptotic state, the cytoskeleton structures were destroyed completely, and cells exhibited a further decrease in surface roughness and an increase in cell stiffness. Given that cellular nano-biophysical properties varied with occurrence of the biological responses, we considered that some of them, such as the roughness, “ridge” (nano-micromorphology) and stiffness (biomechanics) of cell surfaces, should be potential biomarkers for evaluating the efficacy of CA-4 and its analogue for detection of biological responses. As a fast, versatile and quantitative nanometric imaging technique, AFM can accomplish rapid detection of nano-biophysical properties at the single cell level, and this technique may be developed into a new visualization tool for high-throughput screening of CA-4 agents.

We also studied the effect of CA-4 on MCF-7 cells. MTT assays showed that MCF-7 cells were less sensitive to CA-4, as demonstrated by a greater than 50-fold increase in IC_50_ values compared with HepG2 and HeLa cells. Twelve hours of CA-4 treatment also induced G2/M phase arrest in MCF-7 cells; however, apoptosis was not noted, even when the CA-4 exposure time was extended to 48 h. Interestingly, the proportion of polyploidy MCF-7 cells was significantly increased during the prolonged CA-4 treatment. All evidence suggested that the corresponding biological responses in MCF-7 cells treated with CA-4 were different from the sensitive cells. It was reported that CA-4 and its analogue induced autophagy and DNA polyploidy in Caco-2 and CT-26 cells [19]. Our autophagy-related assay showed that CA-4 caused dose-dependent autophagy in MCF-7 cells after inducing G2/M phase arrest. As an antagonistic mechanism of apoptosis, autophagy might contribute to the resistance of MCF-7 cells to CA-4, and apoptosis was triggered in CA-4-treated MCF-7 cells immediately after autophagy. We further investigated the alteration of nano-biophysical characteristics in MCF-7 cells after stimulation with CA-4. Consistent with sensitive cells, the increase in cell stiffness and cytoskeleton rearrangement were also recorded; however, the changes in magnitude of these characteristics were less than that noted in sensitive cells. Grady *et al* reported that nocodazol, a tubulin-dissolving drugs, also induced increased stiffness in tumor cells, and it is consistent with our observation and supports our findings [20]. Particularly noteworthy was the fact that the increased roughness and “ridges” were detected in autophagic MCF-7 cells, totally distinguishing them from the changes in sensitive HepG2 and HeLa cells. These observations indicate that the biological responses induced by CA-4 in MCF-7 cells were very different from those in HepG2 and HeLa cells, which may explain the difference in nano-biophysical changes between CA-4 sensitive and non-sensitive cells. Furthermore, it also suggested that the sensitivity of CA-4 and its analogues to tumor cells might be differentiated by the changes in roughness and ridge structure of the cellular surface. Normal cells have a smooth surface and almost no “ridge” structure, but the surface of tumor cells is not smooth and has obvious “ridge” structure. Accordingly, the roughness and “ridge” structure are independent of each other but have a certain relevance. Based on the results of fluorescence imaging of cytoskeletal proteins (Fig 9), we think that the formation of “ridge” structure is related to the cytoskeleton structure. Moreover, nano-biophysical properties of tumor cells will be induced to undergo a corresponding change, when the cytoskeletal protein F-actin was organized in different ways.

In brief, applying the AFM nano-technique, we first demonstrated that cellular nano-biophysical properties could be used as bio-parameters for evaluating CA-4 agents. The sensitivity of cells to CA-4 varied with the roughness and “ridge” structure of the cell surface, which may be attributed to the different sensitivities of various tumor cells to CA-4. After CA-4 treatment, all tumor cells tended to become stiffer, but the Ra and Rq values exhibited diverse changes. CA-4 decreased Ra and Rq values in sensitive HepG2 and HeLa cells but increased these values in resistant MCF-7 cells. We hypothesize that cellular nano-biophysical properties, such as the roughness and “ridge” structure, could serve as biomarkers to choose candidate tumor cells and/or predict potential mechanisms for CA-4 compounds. Building a corresponding model will be helpful for the development of promising CA-4 compounds. The AFM technique is an ideal tool that provides a new visual and quantitative method to detect the nano-biophysical properties to solve these problems.

## Supporting information

S1 Fig IC50 values for cell growth inhibition by CA-4. The growth inhibitory effects of CA-4 on various tumor cells were measured by MTT assay following 72 h of treatment. IC50 values were calculated using the software of Statistical Product and Service Solutions. Data were shown as mean ± S.D from three independent experiments.
(TIF)

S2 Fig Nanoscale morphology of L929 and U251 cells was performed by AFM. The first column shows full and magnified height image of cells. The second and third columns show full and magnified deflection images of 70×70 μm and 10×10 μm, respectively.
(TIF)
