# Supplementary figures and images for: Nanoscale quantification of the biophysical characterization of combretastatin A-4-treated tumor cells using atomic force microscopy
Source: PLoS One. 2017 Jun 19;12(6):e0179115. doi: 10.1371/journal.pone.0179115 (PMC5476243; doi:10.1371/journal.pone.0179115)

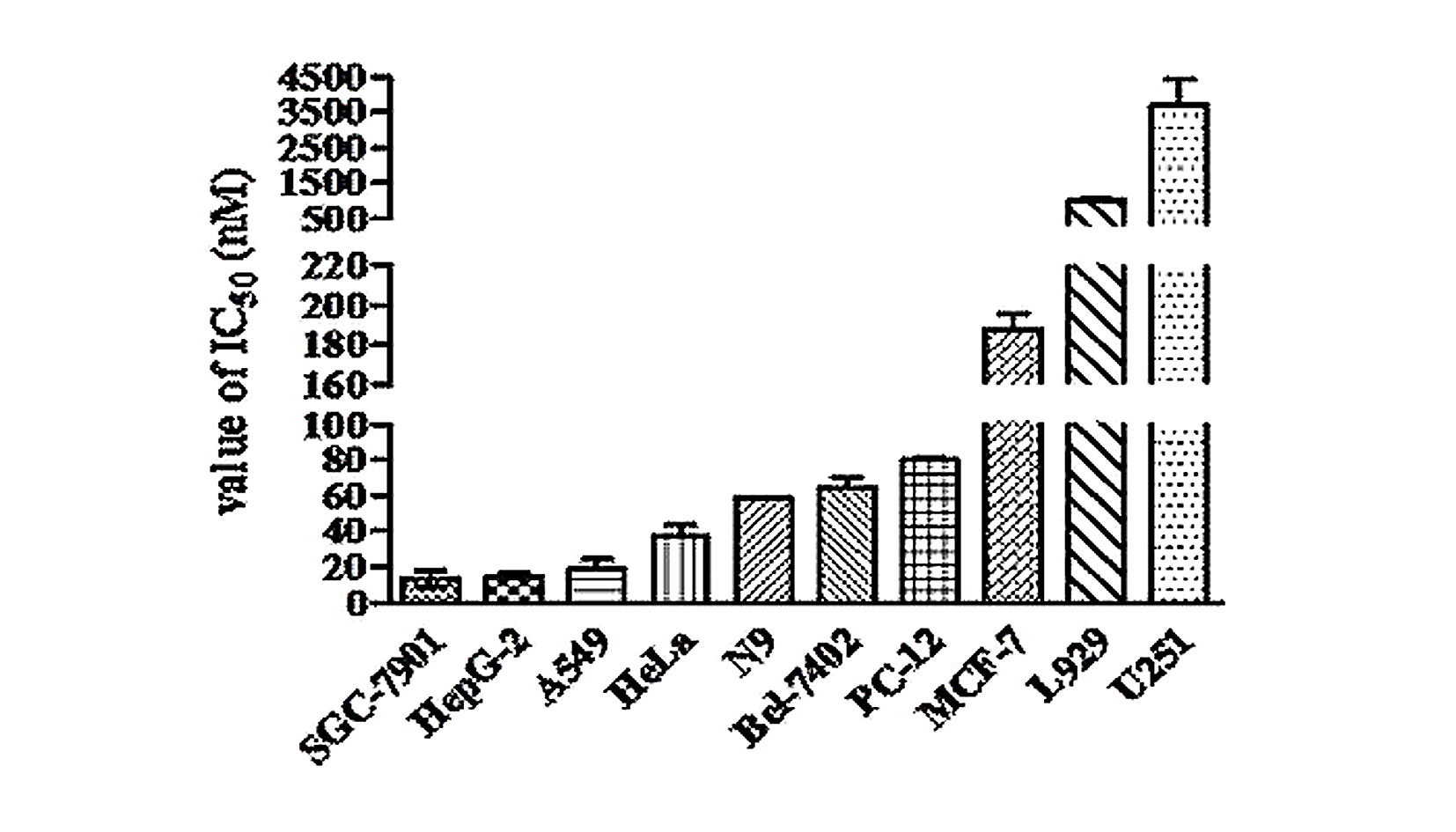

Supplement: S1 Fig — The growth inhibitory effects of CA-4 on various tumor cells were measured by MTT assay following 72 h of treatment. IC50 values were calculated using the software of Statistical Product and Service Solutions. Data were shown as mean ± S.D from three independent experiments. (TIF) [file pone.0179115.s001.tif]

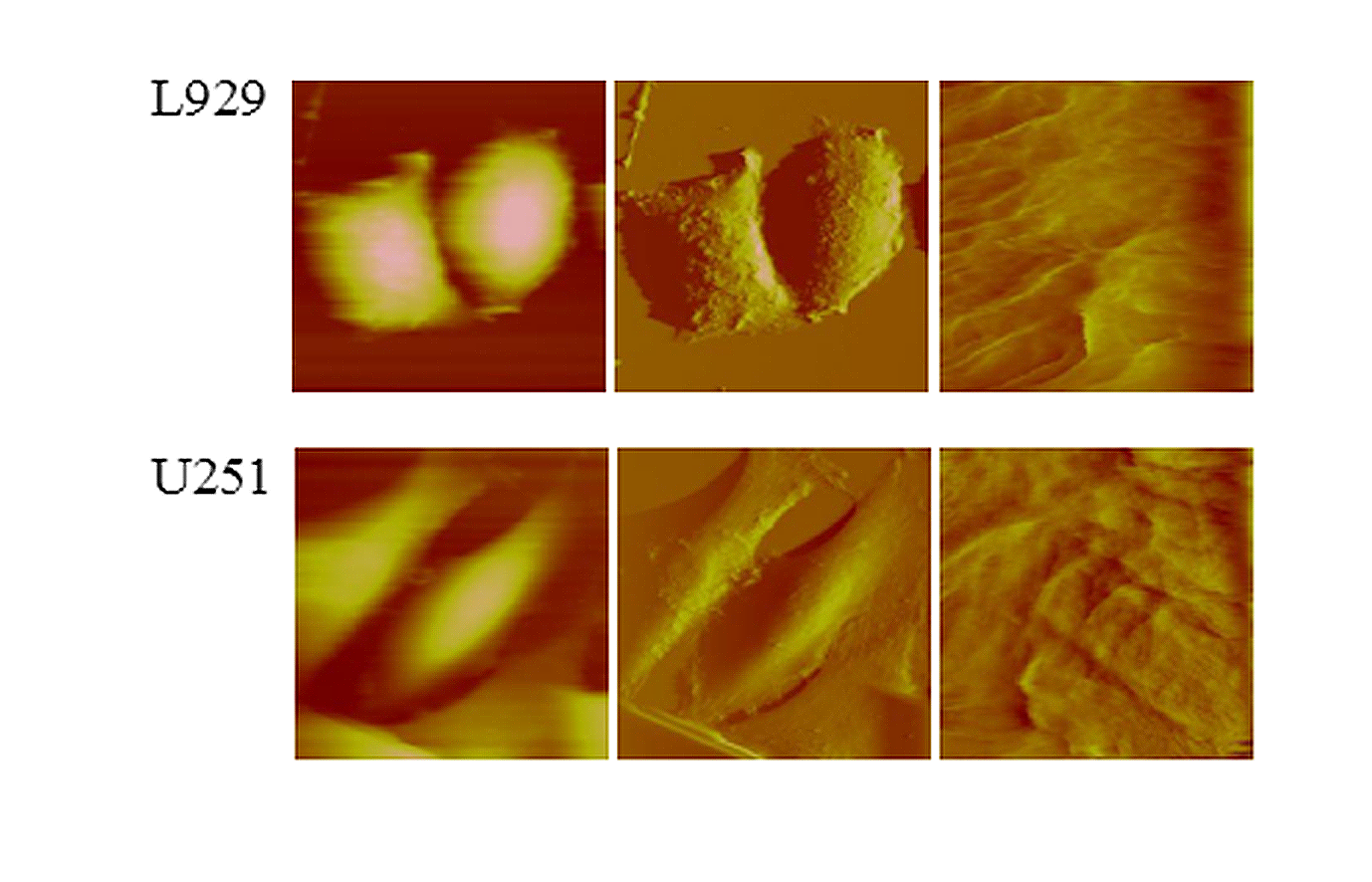

Supplement: S2 Fig — The first column shows full and magnified height image of cells. The second and third columns show full and magnified deflection images of 70×70 μm and 10×10 μm, respectively. (TIF) [file pone.0179115.s002.tif]
